# Hydrogeochemical Characterization and Irrigation Quality Assessment of Shallow Groundwater in the Central-Western Guanzhong Basin, China

**DOI:** 10.3390/ijerph16091492

**Published:** 2019-04-27

**Authors:** Panpan Xu, Wenwen Feng, Hui Qian, Qiying Zhang

**Affiliations:** 1 School of Environmental Science and Engineering, Chang’an University, No. 126 Yanta Road, Xi’an 710054, Shaanxi, China; xupanpan0212@163.com (P.X.); 2017129005@chd.edu.cn (W.F.); 2018029002@chd.edu.cn (Q.Z.); 2 Key Laboratory of Subsurface Hydrology and Ecological Effects in Arid Region of the Ministry of Education, Chang’an University, No. 126 Yanta Road, Xi’an 710054, Shaanxi, China

**Keywords:** groundwater, hydrogeochemistry, quality assessment, irrigation, Guanzhong Basin

## Abstract

Groundwater is the major water resource for the agricultural development of the Guanzhong Basin, China. In this study, a total of 97 groundwater samples (51 from the North Bank of the Wei River (NBWR) and 46 from the South Bank of the Wei River (SBWR)) were collected from the central-western Guanzhong Basin. The aim of this study was to investigate the hydrogeochemical characteristics of the basin and to determine the suitability of shallow groundwater for irrigation. The groundwater of the entire study area is alkaline. The groundwater of the SBWR is fresh water, and the NBWR groundwater is either freshwater or brackish water. The average concentration of ions (except for Ca^2+^) in SBWR samples is lower than in NBWR samples. HCO_3_^−^ is dominant in the groundwater of the study area. Ca^2+^ is dominant in the SBWR while Na^+^ is dominant in the NBWR. The SBWR groundwater is mainly of the HCO_3_-Ca·Mg type, and has undergone the main hydrogeochemical processes of rock weathering-leaching. The hydrochemical facies of the majority of the NBWR groundwater samples are the HCO_3_-Na type with several minor hydrochemical facies of the HCO_3_-Ca·Mg, SO_4_·Cl-Na, and SO_4_·Cl-Ca·Mg types. Its chemistry is mainly controlled by rock weathering, cation exchange, and evaporation. Salinity hazard, sodium percentage, sodium adsorption ratio, residual sodium carbonate, magnesium hazard, permeability index, Kelley’s ratio, potential salinity, synthetic harmful coefficient, and irrigation coefficient were assessed to evaluate the irrigation quality of groundwater. The results of the comprehensive consideration of these indicators indicate that the percentage of NBWR water samples suitable for irrigation purposes ranges between 15.7% and 100% at an average level of 56.7%. Of the SBWR water samples suitable for irrigation, the percentage ranges from 78.3% to 100% with an average of 91.8%. Land irrigated with such water will not be exposed to any alkali hazard, but will suffer from a salinity hazard, which is more severe in the NBWR. Thus, most of the water in the NBWR can be used for soils with good drainage conditions which control salinity.

## 1. Introduction

Groundwater is the predominant water resource and is centrally important for human survival and social development all over the world [1,2,3]. Suitable groundwater resources have been identified as being essential for various uses in support of domestic, agricultural, and industrial development [4,5,6,7,8,9,10,11]. Especially in arid and semi-arid areas, groundwater is more precious for human life, production and development of industry, and agricultural demands due to the limited availability of precipitation and surface water resources [12,13].

The Guanzhong Basin is located on the “Silk Road Economic Belt” and is the political, economic, and cultural center of Shaanxi Province. It occupies an important strategic position in China’s national regional economic pattern [12,14,15]. However, Guanzhong Basin is also an arid and semi-arid area with relatively scarce water resources [14]. Thus, the security of agricultural irrigation water strongly depends on groundwater resources. Relevant research has shown that under the premise of the same type of arable land area, the economic benefit of grain cultivation in irrigated land can reach 2–4 times that in non-irrigated land [16]. To ensure this irrigation benefit of groundwater, it is necessary to precisely identify its quality, which mainly depends upon the content of different ions [17].

The hydrogeochemical characteristics of groundwater reflect the source of the main components, the type of water, its formative mechanism, the water-rock interaction, and the environment of the groundwater reservoir [14,18,19,20]. The physicochemical characteristics of groundwater are also important to determine the effects of natural processes (e.g., the dissolution of minerals, ion exchange, evaporation, and precipitation) and human activities and enable the evaluation of its quality for agricultural irrigation purposes [11,19,21,22,23]. Agricultural irrigation has much higher demands on water quality than industrial and even household water [2]. Various important ions (such as Na^+^, Ca^2+^, Mg^2+^, and K^+^) in groundwater are indispensable for the maintenance of crop growth and development [19]. However, if the concentration of a particular ion exceeds a specific threshold, groundwater irrigation cannot be utilized. For example, excessive Na^+^ and Cl^−^ in water physically and chemically affect plants and soils, which results in a decline in productivity [24]. If soil is salinized, its permeability decreases [25,26]. The osmotic pressure of plant structural cells decreases, thus preventing water from reaching branches and leaves [17]. For example, salinity hazard which can be measured by total dissolved solids (TDS) or electric conductivity (EC) leads to the inability of plant roots to absorb water with high concentrations of salt in soil [22]. Sodium hazard caused by high sodium concentrations can reduce soil permeability and thus inhibit the absorption of water by crops [17]. Magnesium hazard caused by magnesium excess can lead to soil alkalinity, resulting in declining crop yields [27]. The quality of irrigation water can be assessed with various water quality indices that are widely used worldwide. These include: salinity hazard (SH), sodium percentage (Na%), sodium adsorption ratio (SAR), residual sodium carbonate (RSC), magnesium hazard (MH), permeability index (PI), Kelley’s ratio (KR), potential salinity (PS), synthetic harmful coefficient (K), and the irrigation coefficient (K_a_) [17,19,25,28,29,30,31,32].

Groundwater is an essential requirement for the agricultural development of the Guanzhong Basin, especially shallow groundwater [33,34,35]. However, due to continuing socioeconomic development, the demand for groundwater continues to increase as the pollution of surface water becomes increasingly severe [33]. Therefore, the aim of this study is to explore the hydrogeochemical characteristics and formative mechanism of shallow groundwater, and to assess the suitability of its quality for irrigation purposes in the central-western Guanzhong Basin. The results of this study also provide essential information regarding the agricultural usability of groundwater for decision makers.

## 2. Site Description

### 2.1. Geography and Climate

The Guanzhong Basin is located in the center of Shaanxi province, China, at latitudes from 107°30’ to 110°30’ E, and longitudes from 34°00’ to 35°40’ N [36]. It is bounded by the Qinling Mountains to the south, the North Mountains to the north, the Baoji Canyon to the west, and the Tongguan to the east [37]. The Guanzhong Basin occupies 2 × 10^4^ km^2^. It is 700–800 m above sea level (m a.s.l.) in the eastern part and lowers to a minimum height of 325 m a.s.l. on the western edge, with the average elevation of 400 m a.s.l. [36,37]. Xi’an is the Guanzhong urban agglomeration center, and it is a major agricultural production base of Shaanxi Province [38]. The Guanzhong Basin has been part of the most important political, economic, cultural, social, and educational areas and has had the highest population density in northwestern China since ancient times [39].

**Figure 1:**
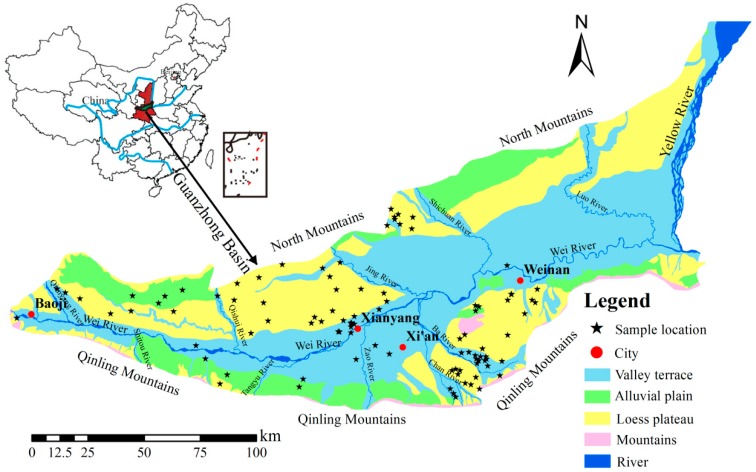
Study area and sampling locations.

The Guanzhong Basin is characterized by a warm temperate continental semi-humid climate with an average annual temperature of 13.3 °C, an average of 1500–2200 h annual daylight and a frost-free period of 200–220 days [40]. Annual precipitation decreases from 850–1000 mm in the Qinling Mountain piedmont to 530–600 mm in the basin center [36]. The annual distribution of precipitation is uneven. The precipitation is mainly concentrated from July to September when approximately 45% of the annual precipitation falls [36,38]. Evaporation generally ranges between 1000 and 1200 mm per year [40]. Many rivers flow through the Guanzhong Basin, including the Yellow River, the Wei River, and the Jing River (Figure 1).

### 2.2. Geomorphological Setting

The general topography of the Guanzhong Basin is high in the west and low in the east, high in the south and north, and low in the middle. The main terrain can be characterized as valley terraces, alluvial plains, and loess plateau (Figure 1). Valley terraces are asymmetrically distributed in the tributaries of the Wei River, and can be divided into five levels. The terraces of the first and second levels are flat, and the terraces of the third to fifth levels are covered with loess at different thickness [39]. The alluvial plain is mainly distributed in the piedmont of the North Mountains and the Qinling Mountains, and consists of deposits of either loess or sediment [41]. The loess plateau is formed by loess that covers the valley terrace with a total thickness of 100–150 m. The lower part is mostly alluvial, lacustrine sand, and gravel at the beginning of the Quaternary, and the upper part is Quaternary aeolian loess with ~20 layers of paleosol [39]. In addition, mountainous areas are scattered in the north of the basin.

### 2.3. Geologic and Hydrogeological Setting

The Guanzhong Basin is a Cenozoic fault basin. The southern boundary is close to the Qinling Mountain fold belt, and the northern boundary is formed by the North Mountains. The Yellow River forms the eastern boundary and isolates the zone of groundwater flow from the Fen River drainage area, making the former an independent hydrogeological unit [36,41]. Subsidence in the basin began during the Late Eocene and continued into the Miocene and Pliocene epochs, leading to the deposition of a thick sequence of fluvial–lacustrine clastic rocks [36]. Aquifers are widely and continuously distributed. Aquifer media is mainly porous and pore-fissure water-bearing rocks in Quaternary loose layers, and often consists of a double-layer aquifer structure of upper phreatic water and lower confined water. The distribution of bedrock fissure water-bearing rocks is relatively small.

The main water-bearing rock formations of the Guanzhong Basin are as follows: the Quaternary alluvial rock group of the valley terrace, the Quaternary alluvial rock group of the alluvial fan, the Quaternary aeolian rock group of the loess plateau, and the limestone rock group of both North Mountains and loess plateau. The groundwater of the Guanzhong Basin is mainly recharged by atmospheric precipitation. The infiltration coefficient of the valley flood plain and first terrace is the highest coefficient, reaching 0.3–0.5. The infiltration coefficient of the second and third terraces is 0.2–0.3, and is 0.1–0.3 for the loess plateau [41]. Furthermore, the permeability of the sediments in the leading and middle edges of the proluvial fan exceeds that of the trailing edge [36]. The rivers on the South Bank of the Wei River (SBWR), which are fed by the Qinling Mountains, have a large amount of water. The aquifer before the Qinling Mountains is an unconsolidated rock layer of Quaternary diluvium with good permeability and high leakage coefficient. Hence, groundwater can be recharged by surface water. For the North Bank of the Wei River (NBWR), the groundwater in the low terrace of floodplain is recharged by precipitation, while the groundwater in the alluvial-diluvial fan areas of rivers and piedmont is recharged by both river side infiltration and atmospheric precipitation infiltration. The flow direction of groundwater is basically consistent with both the topography and groundwater flows from the sides to the center of the basin. The main modes of groundwater drainage are evaporation, horizontal drainage to rivers, spring, and artificial exploitation [41].

## 3. Materials and Methods

### 3.1. Sample Collection and Analysis

To determine the hydrogeochemical characteristics and to assess the water-quality for irrigation, 97 groundwater samples were collected from a phreatic aquifer during a field investigation in the central and western Guanzhong Basin in 1999. Among these, 51 samples were collected from the NBWR, and 46 samples were collected from the SBWR (Figure 1). The water pH and TDS were measured immediately in the field using portable devices on site. EC was calculated from TDS. Furthermore, the name, latitude, and longitude of each sampling point were recorded using portable GPS devices. The cations (K^+^ + Na^+^, Ca^2+^, and Mg^2+^) and anions (HCO_3_^−^, SO_4_^2^^−^, and Cl^−^) were tested in the laboratory. The concentrations of K^+^ + Na^+^ were measured by flame atomic absorption spectrophotometry. Ca^2+^ and Mg^2+^ were analyzed by the EDTA titrimetric method. SO_4_^2^^−^ and Cl^−^ were determined by ion chromatography, and HCO_3_^−^ was tested by alkalinity titration.

After analysis of ion concentrations, the charge balance error (CBE) was calculated to ensure suitably high quality and the standard error for each sample was calculated using formula (1) [25,42]. CBE values with a limit of ± 5% were considered acceptable [43].

(1) $$\mathrm{CBE}=\frac{\sum \mathrm{cations}-\sum \mathrm{anions}}{\sum \mathrm{cations}+\sum \mathrm{anions}}\times 100\%$$

All cations and anions were expressed in meq/L. The calculated results showed that the CBE values of all water samples in this study ranged from −2.43 to 4.54, which corroborated the reliability of this analysis.

### 3.2. Irrigation Water Quality Evaluation

Groundwater is widely used for irrigation in the Guanzhong Basin. The quality of irrigation water is a reflection of its mineral composition and its effect on plants and soil [19]. The chemical composition of irrigation water directly affects plants and agricultural soils, and leads to less productivity [17,19]. Therefore, a water quality assessment for irrigation is very important for thriving agricultural production in the Guanzhong Basin. In this study, irrigation water quality was evaluated by the parameters of the SH, Na%, SAR, RSC, MH, PI, KR, and PS [19,25,28,29]. These indicators were obtained using the following formulas [17,19,25,44,45]:

(2) $$\mathrm{Na}\%=\frac{\left({\mathrm{Na}}^{+}+K^{+}\right)\times 100}{{\mathrm{Ca}}^{2+}+{\mathrm{Mg}}^{2+}+{\mathrm{Na}}^{+}+K^{+}}$$

(3) $$\mathrm{SAR}=\frac{{\mathrm{Na}}^{+}}{\sqrt{\left({\mathrm{Ca}}^{2+}+{\mathrm{Mg}}^{2+}\right)/2}}$$

(4) $$\mathrm{RSC}=\left({\mathrm{CO}}_{3}^{2-}+{\mathrm{HCO}}_{3}^{-}\right)-\left({\mathrm{Ca}}^{2+}+{\mathrm{Mg}}^{2+}\right)$$

(5) $$\mathrm{MH}=\frac{{\mathrm{Mg}}^{2+}}{{\mathrm{Ca}}^{2+}+{\mathrm{Mg}}^{2+}}\times 100$$

(6) $$\mathrm{PI}=\frac{\left({\mathrm{Na}}^{+}+\sqrt{{\mathrm{HCO}}_{3}^{-}}\right)\times 100}{{\mathrm{Ca}}^{2+}+{\mathrm{Mg}}^{2+}+{\mathrm{Na}}^{+}+K^{+}}$$

(7) $$\mathrm{KR}=\frac{{\mathrm{Na}}^{2+}}{{\mathrm{Ca}}^{2+}+{\mathrm{Mg}}^{2+}}$$

(8) $$\mathrm{PS}={\mathrm{Cl}}^{-}+\frac{1}{2}{\mathrm{SO}}_{4}^{2-}$$

where all ionic concentrations are expressed in meq/L.

Moreover, K and K_a_ methods [30] were also applied to evaluate the water quality for irrigation purposes. Their formulas are as follows:

(9) K=12.4M+SAR

(10) $$K_{a}=\{\begin{array}{c} \frac{288}{5{\mathrm{Cl}}^{-}}\;if\;{\mathrm{Na}}^{+}<{\mathrm{Cl}}^{-}\\ \frac{288}{{\mathrm{Na}}^{+}+4{\mathrm{Cl}}^{-}}\;if\;{\mathrm{Cl}}^{-}<{\mathrm{Na}}^{+}<{\mathrm{Cl}}^{-}+2{\mathrm{SO}}_{4}^{2-}\\ \frac{288}{10{\mathrm{Na}}^{+}-5{\mathrm{Cl}}^{-}-9{\mathrm{SO}}_{4}^{2-}}\;if\;{\mathrm{Na}}^{+}>{\mathrm{Cl}}^{-}+2{\mathrm{SO}}_{4}^{2-} \end{array}$$

where M represents the total dissolved solids (in g/L) and SAR represents the sodium adsorption ratio. All ionic concentrations are expressed in meq/L.

## 4. Results and Discussion

### 4.1. Groundwater Chemistry

#### 4.1.1. Physiochemical Parameters

The results of statistical analyses of the physicochemical parameters of NBWR and SBWR groundwater are shown in Table 1. The pH values of NBWR and SBWR groundwater samples ranged from 7.2 to 8.3 (mean = 7.8) and 7.0 to 8.2 (mean = 7.5), respectively. The groundwater in this given area was alkaline. The TDS values of NBWR samples varied in a wide range of 300.0–1324.2 mg/L with a mean of 608.8 mg/L; for SBWR samples, this range was 140.0–692.0 mg/L (mean = 323.5 mg/L). The EC of NBWR and SBWR samples varied at ranges of 545.5–2407.6 μS/cm and 280.0–1258.2 μS/cm, with average values of 1106.8 μS/cm and 588.8 μS/cm, respectively. The higher concentrations of TDS and EC in the NBWR might be due to the more pronounced water-rock interaction, such as the mineral dissolution and evaporation concentration functions.

The major ion contents determine the basic hydrochemical characteristics of groundwater [10]. Overall, as shown in Table 1, the mean values of all ions (except for Ca^2+^) in NBWR samples exceeded those in SBWR samples. K^+^ + Na^+^ of NBWR samples had the highest average concentration (mean = 153.0 mg/L), followed by Ca^2+^ (mean = 37.1 mg/L) and Mg^2+^ (mean = 32.2 mg/L). With regard to the SBWR, the order of cations was Ca^2+^ > K^+^ + Na^+^ > Mg^2+^, with average values of 58.3 mg/L, 38.7 mg/L, and 20.4 mg/L, respectively. Based on the means, the abundance of anions in all samples followed HCO_3_^−^ > SO_4_^2−^ > Cl^-^. Moreover, HCO_3_^−^ in NBWR and SBWR samples ranged from 231.9 to 742.0 mg/L and 121.4 to 482.0 mg/L, with means of 323.3 mg/L and 463.3 mg/L, respectively. The SO_4_^2−^ concentration varied from 2.4 to 416.9 mg/L (NBWR), and from 2.2 to 153.7 mg/L (SBWR). The Cl^−^ concentration varied from 6.0 to 400.6 mg/L (NBWR), and from 2.5 to 122.3 mg/L (SBWR).

#### 4.1.2. Hydrochemical Facies

**Figure 2:**
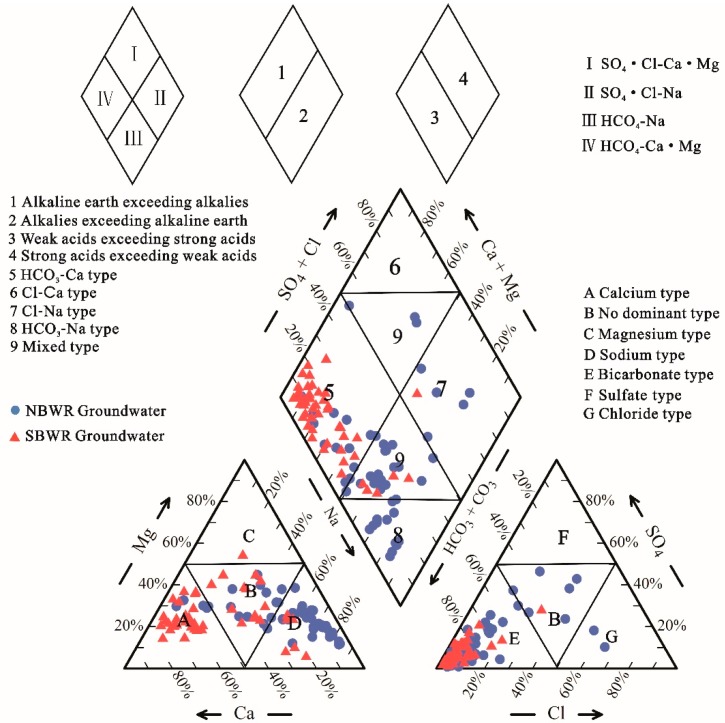
Piper diagram for groundwater samples.

The classification of groundwater can be understood by plotting the major chemical compositions in a Piper diagram [46]. Hydrochemical facies are distinct zones that describe the dominant cations and anions that influence the hydrochemistry of groundwater [17,19,46]. As shown in Figure 2, HCO_3_-Ca·Mg was the dominant water type in SBWR groundwater. This indicates the predominant influence of dissolution on SBWR groundwater chemistry [47]. The water types of the NBWR were relatively complex. HCO_3_-Na was dominant, followed by HCO_3_-Ca·Mg, which can be related to carbonate-rich minerals in aquifers. Moreover, five samples were of SO_4_·Cl-Na type, and three samples were of SO_4_·Cl-Ca·Mg type. Overall, the hydrochemical facies of NBWR samples were mainly influenced by ion exchange, evaporation, and concentration [25,47].

#### 4.1.3. Groundwater Natural Formative Mechanisms

**Figure 3:**
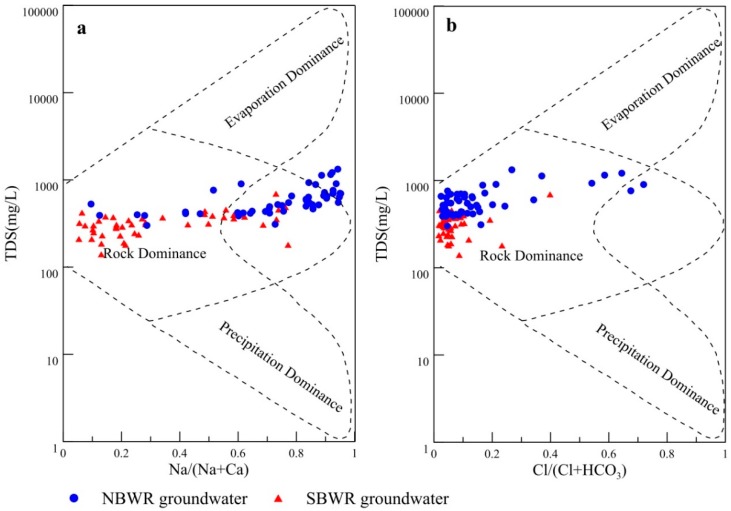
Gibbs diagrams indicating the groundwater natural evolution mechanisms (**a**): TDS vs. Na/(Na+ Ca); (**b**): TDS vs. Cl/(Cl+ HCO_3_).

Gibbs [48] diagrams are helpful to analyze major natural mechanisms that govern groundwater chemistry [13,49]. Gibbs diagrams visualize the relationship between groundwater chemistry and aquifer lithology [22]. Based on these diagrams, three main natural mechanisms can be found: evaporation dominance, rock dominance, and precipitation dominance [48,50,51]. As shown in Figure 3, the groundwater samples of the SBWR were in the zone of rock dominance, which suggests that rock weathering and leaching are major processes affecting groundwater chemistry [25]. This is consistent with hydrogeological conditions. The SBWR is the main recharging area of groundwater in the Guanzhong Basin. The NBWR groundwater samples were also mainly plotted in the rock dominance zone. However, the distribution of these groundwater samples showed a slight increasing trend with the evaporation dominance zone. The main factors governing groundwater chemistry in this area are rock weathering and evaporation. This is primarily due to the lack of precipitation in the NBWR irrigation area, which has increased the intensity of evaporation.

#### 4.1.4. Sources of Major Ions

**Figure 4:**
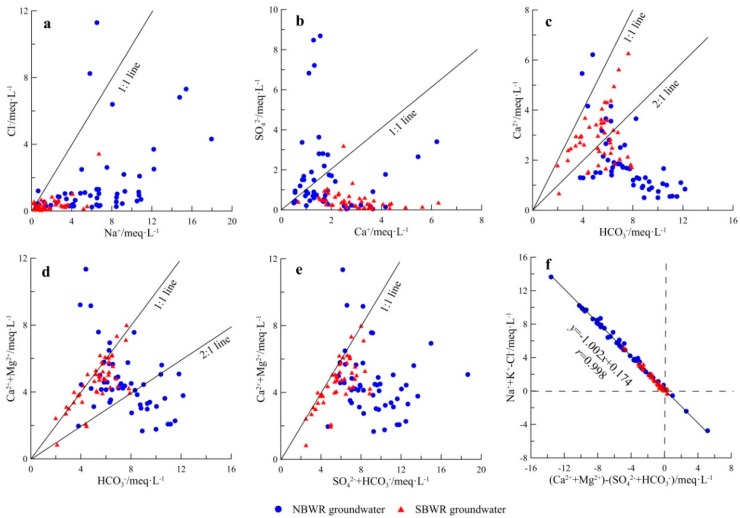
Bivariate diagrams of major ionic concentrations in groundwater samples (**a**): Cl^-^ vs. Na^+^; (**b**): SO_4_^2-^ vs. Ca^2+^; (**c**): HCO_3_^−^ vs. Ca^2+^; (**d**): HCO_3_^−^ vs. Ca^2+^ + Mg^2+^; (**e**): HCO_3_^−^ + SO_4_^2−^ vs. Ca^2+^+Mg^2+^; (f): Na^+^ + K^+^ − Cl^−^ vs. (Ca^2+^ + Mg^2+^) − (HCO_3_^−^+ SO_4_^2−^).

Gibbs diagrams initially indicated the SBWR and NBWR groundwater natural formative mechanisms. Sources of major ions can be further studied by bivariate diagrams of ions [25]. In general, water samples are distributed along the 1:1 line, if Na^+^ and Cl^−^ only come from halite dissolution [52]. The Na^+^ vs. Cl^−^ plot indicated a clear enrichment of Na^+^ in the groundwater samples of both SBWR and NBWR (Figure 4a). This suggests that the cation exchange and the weathering of silicate minerals are responsible for the Na^+^ abundance [10,53,54].

Similarly, the Ca^2+^/ SO_4_^2−^ ratio should be approximately 1 if the dissolution of gypsum would be the only source of Ca^2+^ and SO_4_^2−^ [22,55]. As shown in the bivariate diagram of Ca^2+^ and SO_4_^2−^ (Figure 4b), all groundwater samples of the SBWR were basically below the 1:1 line. This indicates that the dissolution of carbonates, such as calcite and dolomite, may be responsible for the Ca^2+^ excess [42]. Moreover, 64.7% of NBWR groundwater samples also shifted to the left of the 1:1 line. However, for 35.3% of NBWR groundwater samples, the dominance of SO_4_^2−^ over Ca^2+^ indicates that evaporation is responsible for the evolution of water chemistry [25].

The relationship of HCO_3_^-^ with Ca^2+^ and Mg^2+^ involves the dissolution of calcite and dolomite, both of which control the concentrations of these three ions. In theory, the plots should range between 1:1 and 2:1 lines if the dissolution of calcite and dolomite would be the main process [25,50,56]. In Figure 4d, most of the SBWR water samples concentrated in the area of HCO_3_^−^ vs. Ca^2+^ + Mg^2+^ equaling from 1:1 to 2:1, suggesting that dolomite dissolution is the primary source of Ca^2+^ and Mg^2+^. As shown in Figure 4c, approximately 40% of SBWR water samples were plotted below the 1:2 line, suggesting that the effect of calcite dissolution is second. Since the ratio of Na^+^/Cl^-^ exceeds 1, the cation exchange is also responsible for the excess HCO_3_^−^ over Ca^2+^. The ionic characteristics of NBWR water samples are similar to those of the SBWR. However, very few water samples were found above 1:1 (Figure 4c, d), which reflects the influence of gypsum dissolution.

The ratio of HCO_3_^−^ + SO_4_^2−^ vs. Ca^2+^+Mg^2+^ is close to 1 if carbonates (such as calcite and dolomite) and sulfate minerals (such as gypsum) would be the main processes to affect groundwater chemistry [10,57]. In Figure 4e, all groundwater samples of NBWR and SBWR decreased below the 1:1 line. The dominance of HCO_3_^−^ +SO_4_^2−^ over Ca^2+^ + Mg^2+^may be due to silicate weathering and cation exchange. However, only few samples were found above the 1:1 line, indicating that reverse cation exchange plays a controlling role in groundwater. Similarly, the linear relationship of Na^+^ + K^+^ − Cl^−^ vs. (Ca^2+^ + Mg^2+^) − (HCO_3_^−^+ SO_4_^2−^) has a slope of −1 if cation exchange would be the dominant processes in the groundwater [25,58]. The slope of the linear fitting line was −1.002 (Figure 4f), which was very close to −1. This indicates that cation exchange happens in the groundwater system, and that this reaction is more significant in the NBWR groundwater than in the SBWR groundwater. In addition, reverse cation exchange reactions should occur in the NBWR groundwater system.

### 4.2. Groundwater Suitability for Irrigation

Water-quality assessment plays an major role in irrigation practices [17,22]. Groundwater evaluation in the central-western Guanzhong Basin was conducted by using single indexes (SH, Na%, SAR, RSC, MH, PI, KR, and PS), K, and K_a_. These chemically important parameters calculated for irrigation are shown in Table 2.

#### 4.2.1. Salinity Hazard (SH)

In arid and semi-arid areas, the salts in the soil tend to accumulate due to strong evaporation. Consequently, high salinity groundwater forms, so that the roots of plants cannot absorb sufficient water to meet their metabolic requirements [22]. As Alam [59] reported, SH can be judged by the EC. The main lethal effect of a high EC in water is that the failure of plants to compete with ions in the soil results in a physiological condition similar to drought [19,60]. The groundwater classifications based on the salinity hazard are presented in Table 3. The majority of samples (40 samples, 87.0% of all samples) in groundwater of the SBWR were suitable for irrigation, while six samples (13.0% of all samples) had doubtful water quality. For groundwater of the NBWR, the doubtful classification was the main water quality, accounting for 82.4%. 15.7% and 1.9% of all samples were suitable and unsuitable categories, respectively.

#### 4.2.2. Sodium Percentage (Na%)

Na% is an indicator of sodium hazard. A high Na% in soil can have devastating impacts on soil structure, aeration, and infiltration [29,61]. In Table 2, Na% of both NBWR and SBWR groundwater ranged from 6.7% to 83.8% and from 3.7% to 72.9%, respectively. The corresponding mean values were 56.0% and 24.7%, respectively. The samples with permissible water quality and better accounted for 52.9% and 93.5% for NBWR and SBWR groundwater, respectively (Table 4).

**Figure 5:**
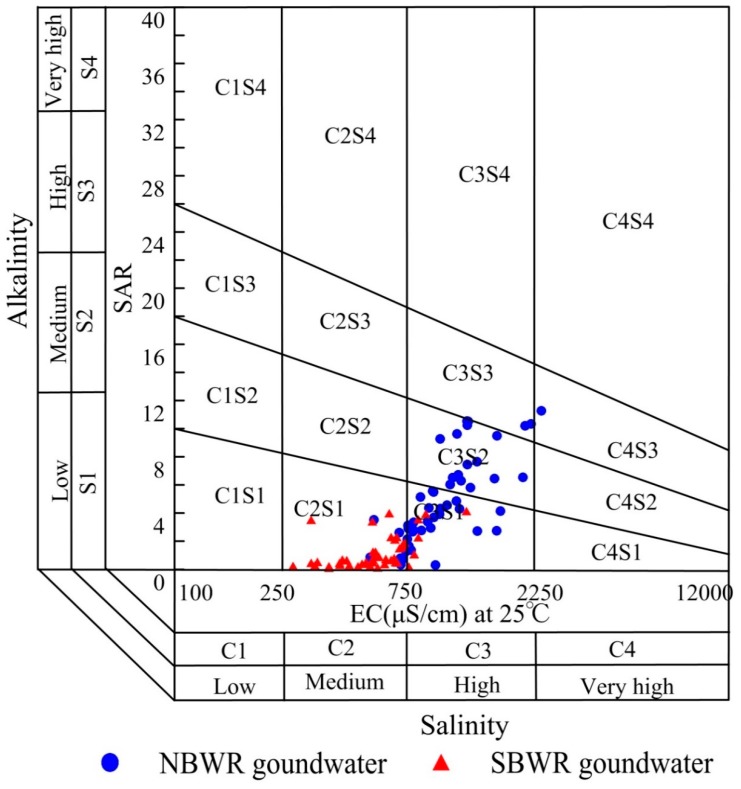
Wilcox diagram for irrigation water quality assessment.

A Wilcox diagram [25,62] was used to assess the suitability of groundwater for irrigation. In Figure 5, the majority of the SBWR groundwater samples were categorized as excellent to good, indicating that the SBWR groundwater is perfect for irrigation. For the NBWR groundwater, 45.1% and 7.8% of samples were categorized as permissible to doubtful and doubtful to unsuitable, respectively. This suggests poor groundwater suitability for irrigation. The most likely reason for such a high level of sodium in the NBWR groundwater is the strong interaction of water and rock, which leads to cation exchange in particular. Additionally, agricultural activities, such as the use of agrochemicals, can also increase the sodium content [19].

#### 4.2.3. Sodium Adsorption Ratio (SAR)

The SAR is also a characterization of sodium hazards, which can reduce soil permeability and thus inhibit the absorption of water by crops [17]. The following groundwater classifications are based on SAR: excellent (SAR < 10), good (10 < SAR < 18), doubtful (18 < SAR < 26), and unsuitable (SAR > 26) [27]. SAR of NBWR groundwater samples varied from 0.3 to 11.3 with a mean of 4.9, while SBWR groundwater samples ranged from 0.1 to 4.1 with a mean of 1.2.

**Figure 6:**
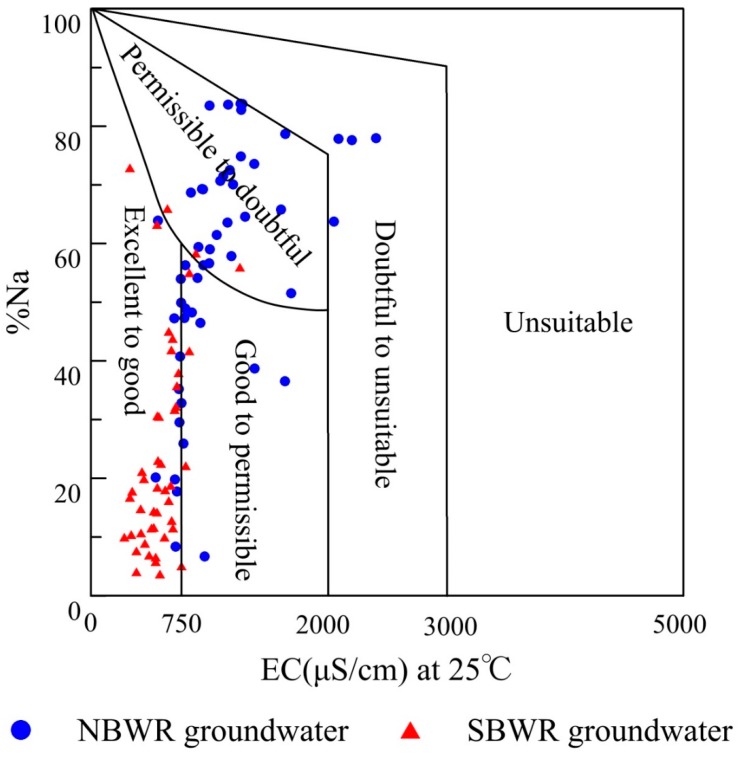
USSL diagram for assessing irrigation water quality.

Deeper insight into the suitability of water for irrigation can be obtained with plotting the SAR against the EC using a U.S. Salinity Laboratory (USSL) diagram [63]. According to Figure 6, about 87.0% of the SBWR samples were classified as C2S1, indicating medium salinity/low sodium. Hence, it is suitable for irrigation. Only 13.0% of samples were classified as C3S1 indicating high salinity/low sodium, which suggests that this groundwater is not suitable for irrigation due to its high salinity. The NBWR samples were mainly classified as high salinity categories (C3S1 and C3S2). Thus, most of the water in the northern part of the study area can be used to irrigate soils with good drainage conditions, which control salinity [29,64].

#### 4.2.4. Residual Sodium Carbonate (RSC)

Bicarbonate is an important component for evaluating the quality of irrigation water [25]. RSC is a valuable tool for examining the applicability of irrigation water by measuring the relationship between the sum of carbonate and bicarbonate and the sum of calcium and magnesium [19,29,65]. Soils irrigated with high RSC water can become infertile due to deposition of sodium carbonate [25,66].

The RSC of NBWR groundwater samples ranged from −6.9 to 9.3 meq/L with an average of 3.1 meq/L, and the RSC of SBWR groundwater ranged from −0.7 to 4.0 meq/L with an average of 0.7 meq/L (Table 2). Table 5 shows the groundwater classifications according to the RSC in the study area. In the SBWR groundwater, 80.4% of samples were classified as good water, indicating suitability for irrigation. However, 13.1% and 6.5% of samples were classified as doubtful and unsuitable, respectively. 19.6% and 54.9% of the NBWR samples were classified as doubtful and unsuitable water type, respectively, and only 25.5% of samples were classified as good for irrigation purposes. This may be due to the widespread distribution of Quaternary sediments in both the valley terrace and the loess plateau, as these are rich in calcite and dolomite and leach substantial amounts of HCO_3_^−^.

#### 4.2.5. Magnesium Hazard (MH)

MH indicates the degree of damage to the soil structure caused by magnesium in irrigation water [17]. A high level of Mg^2+^ in groundwater leads to soil alkalinity; furthermore, a large amount of water is adsorbed between magnesium and clay particles, which reduces the infiltration capability of soil, which has adverse effects on crops [19,27,68]. A value of MH > 50 indicates harmful groundwater and unsuitable for irrigation, while a value of MH < 50 indicates suitable groundwater [32].

The MH of NBWR and SBWR samples ranged from 32.1 to 80.2% (mean = 60.7%) and from 15.8 to 69.9% (mean = 35.5%), respectively (Table 2). Furthermore, 12 (23.5% of the total samples) and 36 (78.3% of the total samples) samples had MH values below 50% for NBWR and SBWR areas, respectively.

#### 4.2.6. Permeability Index (PI)

Long-term use of minerally rich (Ca^2+^, Mg^2+^, Na^+^, and HCO_3_^−^) groundwater can reduce aeration in the soil and obstruct the growth of seedlings [19]. The PI is also used to reflect the applicability of groundwater for irrigation purpose [69]. A PI of 75% or above max permeability indicates that the groundwater is suitable for irrigation (class I and class II), while a PI of 25% or below max permeability is regarded as unsuitable for irrigation (class III) [45,70]. Thus, a Doneen [45] diagram was developed using the total concentration of salts and the PI.

**Figure 7:**
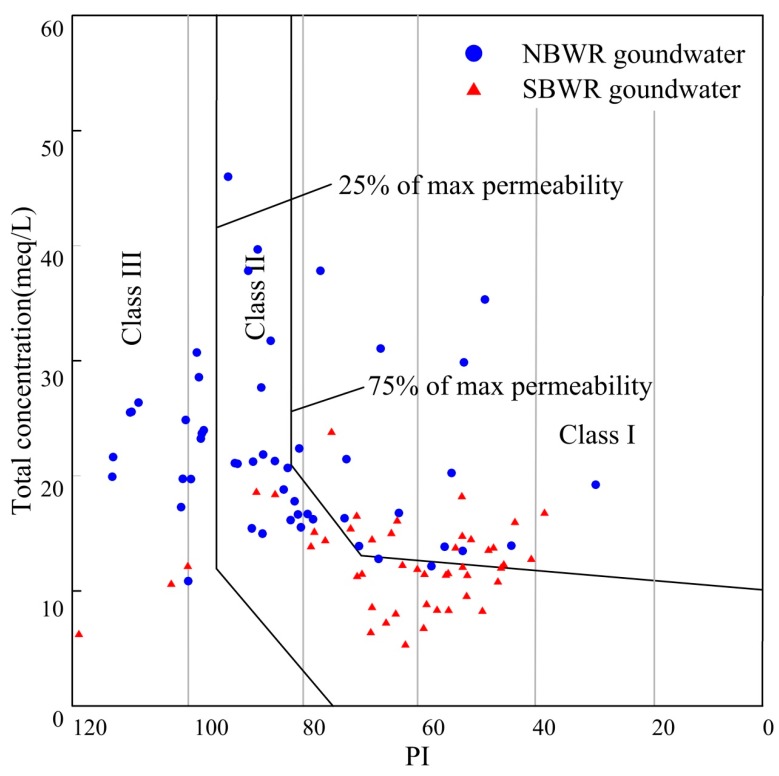
Doneen diagram for assessing irrigation water quality.

The PI of NBWR groundwater samples ranged from 29.0% to 113.0% with an average value of 82.6%. The values of SBWR samples differed and ranged from 37.9% to 118.8% with an average value of 62.8%. Analytical data of PI values are presented as a Doneen diagram. Figure 7 shows that 93.5% of the SBWR groundwater samples were classified as class I and class II, and 6.5% were classified as class III. With regard to NBWR groundwater, 70.6% of samples were suitable for irrigation, and the remaining 29.4% were unsuitable for irrigation. High PI values are related to high levels of Na^+^ and HCO_3_^−^, which may be due to the cation exchange and carbonate dissolution (such as calcite and dolomite).

#### 4.2.7. Kelley’s Ratio (KR)

The suitability of groundwater for irrigation can also be assessed with KR [25]. The KR of NBWR water samples ranged from 0.1 to 5.2, and that for SBWR water samples ranged from 0.1 to 2.7. Furthermore, their corresponding average levels were 1.8 and 0.5, respectively. If KR < 1, the water is suitable for irrigation, otherwise, it is not suitable for irrigation [71]. Thus, approximately 87.0% of the samples from SBWR groundwater were within an acceptable level, and 13.0% of samples did not meet the irrigation requirements. However, 64.7% of the NBWR groundwater samples were unsuitable for irrigation, which may also be due to intense cation exchange, which provides excess Na^+^.

#### 4.2.8. Potential Salinity (PS)

PS is defined as the Cl^-^ concentration plus half of the SO_4_^2−^ concentration, which can also indicate suitability of groundwater for irrigation [17]. The groundwater quality classifications based on the PS are listed in Table 6. The values of PS in the NBWR water samples varied from 0.3 to 12.2 meq/L (mean = 2.5 meq/L). 80.4% of samples were classified between excellent and good. 5.9% and 13.7% of samples were classified as good to injurious and the injurious to unsatisfactory class, respectively. For SBWR water samples, PS values ranged from 0.1 to 5.0 meq/L (mean = 0.6 meq/L). 97.8% of samples were classified as excellent to good, and only 2.2% of samples classified as injurious to unsatisfactory, indicating that the groundwater in the SBWR has high suitability for irrigation.

#### 4.2.9. Synthetic Harmful Coefficient (K)

K can comprehensively reflect the salt and alkali hazards. Based on K values, the groundwater for irrigation is classified as follows: excellent (K < 25), good (25 < K < 36), injurious (36 < K < 44), and unsuitable (K > 44) [30]. In Table 2, the K values of NBWR groundwater samples ranged from 4.6 to 27.7 with a mean of 12.4. 96.1% of samples were classified as excellent, and 3.9% were classified as good. For SBWR groundwater, the K values ranged from 2.0 to 12.7 with a mean of 5.2, and all the samples were classified as excellent. The evaluation results of this method are conservative, which presents the water quality in a relatively good light.

#### 4.2.10. Irrigation Coefficient (K_a_)

K_a_ can be obtained by studying the strongest harm of alkaline solution and the relative harm of sodium salt to 40 types of crops [30]. The results of this classification of the quality of groundwater used for irrigation are presented in Table 7. The distribution of the groundwater quality in water samples of the NBWR was as follows: 13.7% were classified as excellent, 49.0% were classified as permissible, and 37.3% were classified as doubtful. Values ranged from 3.0 to 120.4 (mean of 13.0), indicating that the average level was permissible. Such groundwater can be used for irrigation; however, action should be taken to avoid salt accumulation. 71.7% and 28.3% of the SBWR water samples were classified as excellent and permissible, respectively. The K_a_ values ranged from 6.4 to 422.1 (mean of 94.5), indicating that the average level was excellent.

## 5. Conclusions

Groundwater plays a decisive role in agricultural development, particularly in arid and semi-arid areas. Hence, its quality and suitability for irrigation is significance. In the current study, the main ions of shallow groundwater were analyzed for an improved hydrogeochemical characterization and irrigation quality assessment of the central-western Guanzhong Basin. The following conclusions can be drawn.

The major groundwater type of the SBWR is HCO_3_-Ca·Mg. The types of groundwater in the NBWR are more complex, and primarily consist of HCO_3_-Na, followed by HCO_3_-Ca·Mg, SO_4_·Cl-Na, and SO_4_·Cl-Ca·Mg. The Na^+^ content in the NBWR exceeds that of the SBWR, which is mainly the result of stronger cation exchange in the NBWR. This is likely the reason for the low Ca^2+^ level in the NBWR. In addition, the high concentrations of Cl^-^ and SO_4_^2-^ in several of the NBWR water samples are responsible for evaporation, followed by human activities. With regard to the groundwater in the SBWR, excess Ca^2+^ is primarily caused by the dissolution of dolomite and calcite. Dolomite leaching also provides sufficient Mg^2+^.

According to the indexes SH, Na%, SAR, RSC, MH, PI, KR, and PS, K, and K_a_, approximately 15.7%, 52.9%, 100%, 25.5%, 23.5%, 70.6%, 35.5%, 80.4%, 100%, and 62.7% of groundwater sources are suitable for irrigation in the NBWR, respectively. Similarly, 87.0%, 93.5%, 100%, 80.4%, 78.3%, 93.5%, 87.0%, 97.8%, 100% and 100% of SBWR groundwater samples are suitable for irrigation, respectively. The results of a comprehensive consideration of these indicators show that the average level of SBWR groundwater samples (91.8%) that are suitable for irrigation purposes far exceeds that (56.7%) of the NBWR. Land irrigated with such water will not be exposed to alkali hazard, but will suffer from SH, which is more severe in the NBWR. Therefore, when using local groundwater (which is in fact brackish water) to irrigate farmland, reasonable measures should be taken to control the salt content in the water and to prevent the accumulation of soil salt.

The results of this study provide guidance for decision makers for the management of groundwater for irrigation purposes in the central-western Guanzhong Basin. However, the limitations of this study are that it does not apply boron or metals to evaluate the water quality for agricultural use; therefore, further research is required.

## Figures and Tables

**Table 1:** Physicochemical groundwater characteristics.

Index	Unit	NBWR Groundwater			SBWR Groundwater		
		Maximum	Minimum	Mean	Maximum	Minimum	Mean
K^+^ + Na^+^	mg/L	412.9	13.7	153.0	154.6	3.9	38.7
Ca^2+^	mg/L	124.2	10.0	37.1	125.5	13.6	58.3
Mg^2+^	mg/L	86.2	7.9	32.2	59.7	2.1	20.4
Cl^−^	mg/L	400.6	6.0	60.4	122.3	2.5	14.0
SO_4_^2+^	mg/L	416.9	2.4	80.2	153.7	2.2	22.6
HCO_3_^−^	mg/L	742.0	231.9	463.3	482.0	121.4	323.3
pH	-	8.3	7.2	7.8	8.2	7.0	7.5
TDS	mg/L	1324.2	300.0	608.8	692.0	140.0	323.5
EC	μS/cm	2407.6	545.5	1106.8	1258.2	280.0	588.8

**Table 2:** Water-quality parameters for irrigation.

Parameter	Unit	NBWR Groundwater			SBWR Groundwater		
		Maximum	Minimum	Mean	Maximum	Minimum	Mean
SH (EC)	μS/cm	2407.6	545.5	1106.8	1258.2	280.0	588.8
Na%	%	83.8	6.7	56.0	72.9	3.7	24.7
SAR	-	11.3	0.3	4.9	4.1	0.1	1.2
RSC	meq/L	9.3	−6.9	3.1	4.0	−0.7	0.7
MH	%	80.2	32.1	60.7	69.9	15.8	35.5
PI	%	113.0	29.0	82.6	118.8	37.9	62.8
KR	-	5.2	0.1	1.8	2.7	0.1	0.5
PS	meq/L	12.2	0.3	2.5	5.0	0.1	0.6
K	-	27.7	4.6	12.4	12.7	2.0	5.2
K_a_	-	120.4	3.0	13.0	422.1	6.4	94.5

NBWR: North Bank of the Wei River; SBWR: South Bank of the Wei River; SH (EC): salinity hazard (electric conductivity); Na%: sodium percentage; SAR: sodium adsorption ratio; RSC: residual sodium carbonate; MH: magnesium hazard; PI: permeability index; KR: Kelley’s ratio; PS: potential salinity; K: synthetic harmful coefficient; K_a_: the irrigation coefficient.

**Table 3:** Groundwater classification quality for irrigation based on the salinity hazard [17].

SH Class	EC (μS/cm)	Water Quality	No. of Samples (Percent)	
			NBWR Groundwater	SBWR Groundwater
C1	<250	Excellent	-	-
C2	250–750	Good	8 (15.7%)	40 (87.0%)
C3	750–2250	Doubtful	42 (82.4%)	6 (13.0%)
C4	>2250	Unsuitable	1 (1.9%)	-

**Table 4:** The classifications of groundwater quality for irrigation based on the Na% [62].

Na%	Water Quality	No. of Samples (Percent)	
		NBWR Groundwater	SBWR Groundwater
<20	Excellent	4 (7.8%)	26 (56.5%)
20–40	Good	7 (13.7%)	10 (21.7%)
40–60	Permissible	16 (31.4%)	7 (15.3%)
60–80	Doubtful	19 (37.3%)	3 (6.5%)
>80	Unsuitable	5 (9.8%)	-

**Table 5:** The classifications of groundwater quality for irrigation based on the RSC (residual sodium carbonate) [67].

RSC (meq/L)	Water Quality	No. of Samples (Percent)	
		NBWR Groundwater	SBWR Groundwater
<1.25	Good	13 (25.5%)	37 (80.4%)
1.25–2.50	Doubtful	10 (19.6%)	6 (13.1%)
>2.50	Unsuitable	28 (54.9%)	3 (6.5%)

**Table 6:** The classifications of groundwater quality for irrigation based on the PS (potential salinity) [72].

PS (meq/L)	Water Quality	No. of Samples (Percent)	
		NBWR Groundwater	SBWR Groundwater
<3.0	Excellent to good	41 (80.4%)	45 (97.8%)
3.0–5.0	Good to injurious	3 (5.9%)	-
>5.0	Injurious to unsatisfactory	7 (13.7%)	1 (2.2%)

**Table 7:** The classifications of groundwater quality for irrigation based on the K_a_ (the irrigation coefficient) [30].

K_a_	Water Quality	No. of samples (Percent)	
		NBWR Groundwater	SBWR Groundwater
>18	Excellent	7 (13.7%)	33 (71.7%)
6–18	Permissible	25 (49.0%)	13 (28.3%)
1.2–6	Doubtful	19 (37.3%)	-
<1.2	Unsuitable	-	-
